# Abnormal Expression of the Pre-mRNA Splicing Regulators SRSF1, SRSF2, SRPK1 and SRPK2 in Non Small Cell Lung Carcinoma

**DOI:** 10.1371/journal.pone.0046539

**Published:** 2012-10-10

**Authors:** Stephanie Gout, Elisabeth Brambilla, Asma Boudria, Romain Drissi, Sylvie Lantuejoul, Sylvie Gazzeri, Beatrice Eymin

**Affiliations:** 1 Equipe 2 Bases Moléculaires de la Progression des Cancers du Poumon, INSERM, U823, Grenoble, France; 2 Institut Albert Bonniot, Université Joseph Fourier, Grenoble, France; 3 Département de Pathologie, Centre Hospitalier Universitaire, Grenoble, France; IPO, Inst Port Oncology, Portugal

## Abstract

Splicing abnormalities frequently occur in cancer. A key role as splice site choice regulator is played by the members of the SR (Ser/Arg-rich) family of proteins. We recently demonstrated that SRSF2 is involved in cisplatin-mediated apoptosis of human lung carcinoma cell lines. In this study, by using immunohistochemistry, we demonstrate that the SR proteins SRSF1 and SRSF2 are overexpressed in 63% and 65% of lung adenocarcinoma (ADC) as well as in 68% and 91% of squamous cell lung carcinoma (SCC), respectively, compared to normal lung epithelial cells. In addition, we show that SRSF2 overexpression correlates with high level of phosphorylated SRSF2 in both ADC (p<0.0001) and SCC (p = 0.02), indicating that SRSF2 mostly accumulates under a phosphorylated form in lung tumors. Consistently, we further show that the SR-phosphorylating kinases SRPK1 and SRPK2 are upregulated in 92% and 94% of ADC as well as in 72% and 68% of SCC, respectively. P-SRSF2 and SRPK2 scores are correlated in ADC (p = 0.01). Using lung adenocarcinoma cell lines, we demonstrate that SRSF1 overexpression leads to a more invasive phenotype, evidenced by activation of PI3K/AKT and p42/44MAPK signaling pathways, increased growth capacity in soft agar, acquisition of mesenchymal markers such as E cadherin loss, vimentin and fibronectin gain, and increased resistance to chemotherapies. Finally, we provide evidence that high levels of SRSF1 and P-SRSF2 proteins are associated with extensive stage (III–IV) in ADC. Taken together, these results indicate that a global deregulation of pre-mRNA splicing regulators occurs during lung tumorigenesis and does not predict same outcome in both Non Small Cell Lung Carcinoma histological sub-types, likely contributing to a more aggressive phenotype in adenocarcinoma.

## Introduction

More than 90% of human genes undergo pre-mRNA alternative splicing leading to the synthesis of various protein isoforms with different biological properties [1] Splicing defects of critical genes have been causally linked to various diseases, including cancer [2]–[4]. Lung cancer is the most common cause of mortality among all cancers, accounting for an estimated 1.3 million deaths worldwide annually. Importantly, a genome wide analysis of alternative splicing events previously demonstrated that a large number of known oncogenes and tumor suppressor genes are alternatively spliced and differentially expressed in lung adenocarcinoma, compared to normal lung [5]. In this histological subtype, it was recently shown that of 5183 profiled alternative exons, four displayed tumor-associated changes in the majority of the patients, namely VEGF-A, MACF1, APP and NUMB genes [6]. Moreover, we and others have reported splicing modifications of genes such as caspases, Bcl-x, CD44, FLIP, MDM2 and VEGF-A in primary lung cancer and cell lines [7]–[14]. Therefore, widespread alternative splicing changes occur in lung cancer and impact cell signaling in a manner that likely contributes to tumorigenesis.

**Table 1:** Demographics and clinical characteristics of the patients.

	Histology	
Demographics and Clinical parameters	ADC (%)	SCC (%)
Median age ± SD (years)	61±10	64±10
Male	41 (76)	52 (98)
Female	13 (24)	1 (2)
Stage I	26 (48)	12 (22)
Stage II	11 (21)	10 (19)
Stage III	10 (18)	27 (51)
Stage IV	7 (13)	4 (8)
pT1	16 (30)	10 (19)
pT2	26 (48)	18 (34)
pT3	7 (13)	13 (24)
pT4	5 (9)	12 (23)
N0	35 (65)	15 (29)
N1	11 (20)	24 (45)
N2	8 (15)	14 (26)

Abbreviations: ADC, adenocarcinoma; SCC, squamous cell carcinoma; SD, standard deviation.

The mechanisms leading to aberrant alternative splicing in cancer are poorly understood. Splicing modifications are associated with cis-acting mutations that affect alternative splice sites. Furthermore, it has been proposed that abnormal expression and/or activity of trans-splicing regulatory proteins mainly contribute to the abnormal alternative splicing patterns detected in tumors [15]. The SR protein family is one of the most important classes of splicing regulators that display essential roles in the enhancement of constitutive and alternative pre-mRNA splicing, as well as in other aspects of gene expression [16]–[19]. Activity of SR proteins is highly regulated by extensive and reversible phosphorylation of serine residues. These phosphorylations modulate protein–protein interactions within the spliceosome [20] and regulate the activity and sub-cellular distribution of SR proteins [21]. Therefore, changes in the phosphorylation state of SR proteins play a critical role in the control of their activity. Several kinases that phosphorylate SR proteins have been identified. They include the SR protein kinases (SRPKs) 1 and 2, the CLK/STY, the DNA topoisomerase I and AKT [21]–[24]. To date, only a few studies have investigated the status of SR proteins in human lung tumors. It was recently shown that the SR protein SRSF1 (former SF2/ASF), previously described as an oncogene [25], is overexpressed in primary non small cell lung carcinoma and controls the expression of survivin, an anti-apoptotic protein [26]. Nothing is known about the status of phosphorylated SR proteins or SRPK kinases in lung cancer. Interestingly, we recently demonstrated that phosphorylation of SRSF2 (former SC35) by SRPK2 is involved in cisplatin-mediated apoptosis of human lung carcinoma cell lines [10].

**Figure 1:**
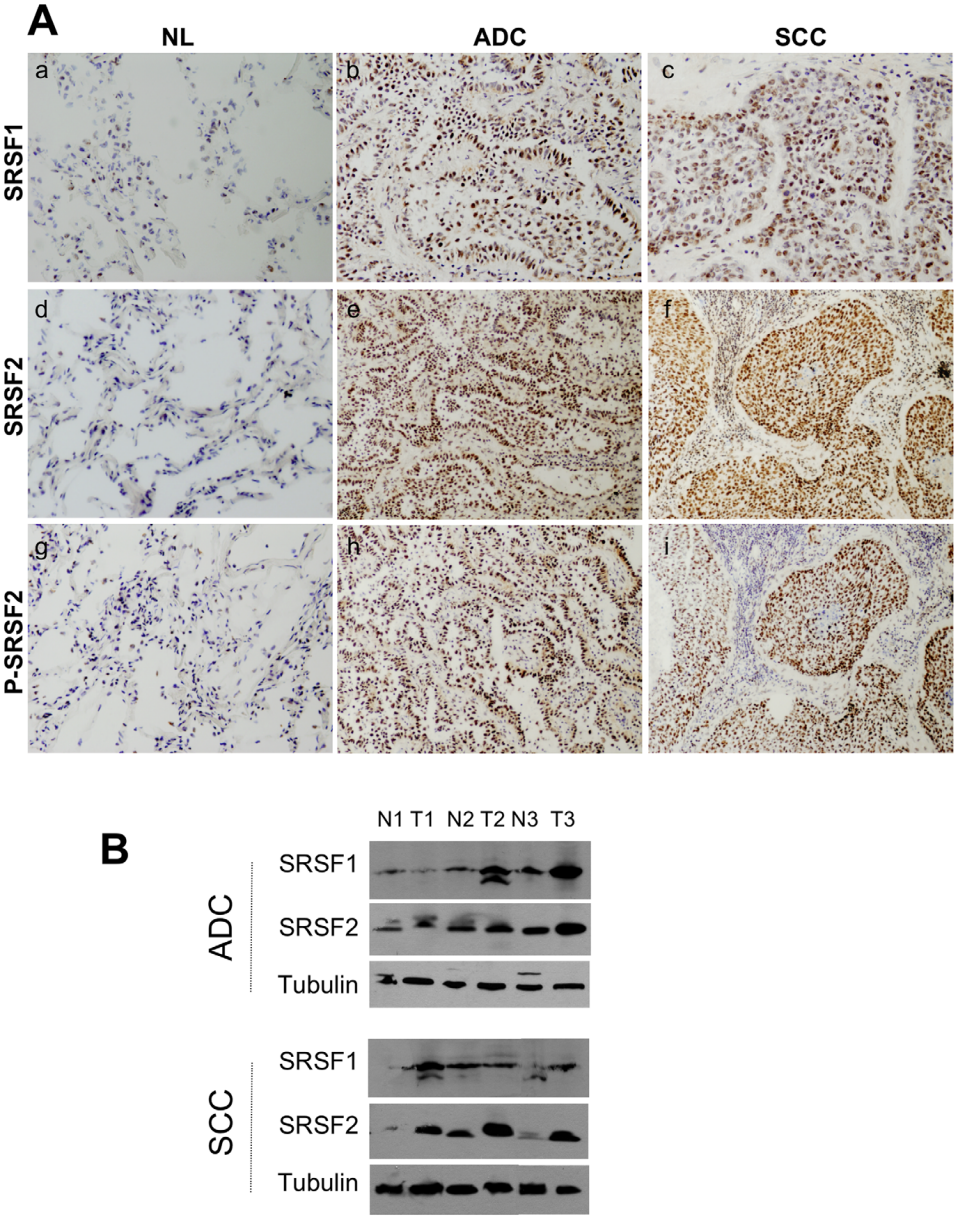
Expression of SRSF1, SRSF2 and phosphorylated SRSF2 proteins in NSCLCs. A, Representative immunostaining from frozen section of normal lung parenchyma and lung cancer tissue with anti SRSF1 (a, b, c), anti SRSF2 (d,e,f) and anti phospho-SRSF2 (g, h, i) antibodies [(a, d, g) normal lung; (b, e, h) ADC; (c, f, i) SCC; immunoperoxidase and haematoxylin counterstaining]. B, Representative western blots demonstrating overexpression of SRSF1 and SRSF2 proteins in lung tumors compared with their matched normal lung tissues. (NL, normal lung; ADC, adenocarcinoma; SCC, squamous cell carcinoma).

**Table 2:** Immunohistochemical analysis of SRSF1 and SRSF2 proteins expression in non-small cell lung cancer according to histological subtype.

	SRSF1 (%)		SRSF2 (%)		Phospho-SRSF2 (%)			
	Class 0	Class 1	Class 0	Class 1	Class 0	Class 1	Class 2	P value
**ADC**	20(37)	34(63)	19(35)	35(65)	14(26)	12(22)	28(52)	<0.0001
**SCC**	17(32)	36(68)	5(9)	48(91)	11(21)	8(15)	34(64)	<0.0001
**NSCLC**	37(35)	70(65)	24(22)	83(78)	25(23)	20(19)	62(58)	<0.0001

Abbreviations: ADC, adenocarcinoma; SCC, squamous cell carcinoma; NSCLC, non-small cell lung carcinoma. Immunostaining scores were calculated by multiplying the number of labeled cells (0–100%) by the level of intensity (1–3). According to this, tumor samples were grouped into several classes (see ‘Materials and Methods’ section). For SRSF1 and SRSF2, class 0 (normal expression as compared to normal lung), class 1 (high staining considered as overexpression). For phospho-SRSF2, class 0 (normal expression), class 1 (moderate overexpression) and class 2 (high overexpression). Statistical analysis was done using Fisher’s exact test.

In this study, we investigated the status of SRSF1, SRSF2 and its phosphorylated form P-SRSF2, as well as of SRPK1 and SRPK2 in a series of 107 NSCLC, including 54 adenocarcinoma (ADC) and 53 squamous cell carcinoma (SCC). Our results reveal a global overexpression of these splicing regulators in NSCLC compared to normal lung tissues, that correlate with more aggressive clinico-pathological features in ADC. In agreement with these data, we provide evidence that overexpression of SRSF1 in cellular models derived from human lung adenocarcinoma leads to a more aggressive phenotype with activation of p42/44MAPK and AKT signaling pathways, increased colony formation in soft agar, epithelial to mesenchymal transition and resistance to carboplatin and paclitaxel.

**Figure 2:**
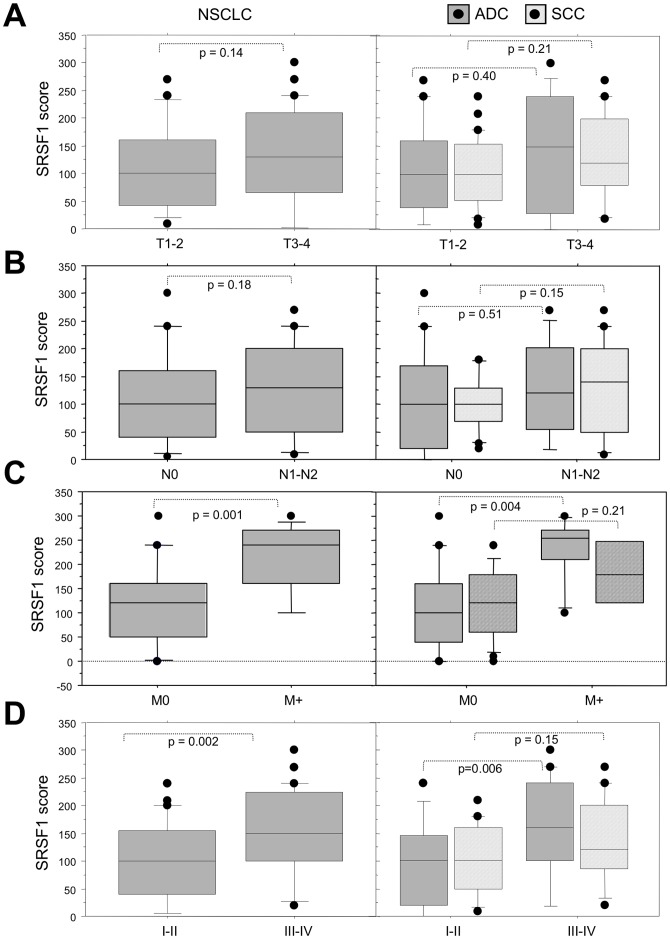
SRSF1 scores according to the clinico-pathological parameters in NSCLC subtypes. Distribution of SRSF1 scores according to the tumor size (A), the nodal status (B), the presence of metastases at distance (C) and the pTNM stage (D), in all the tumors (left panels, NSCLC) and in histological subtypes (right panels, ADC and SCC). Statistical analysis was done using Mann-Whitney’s U test.

## Materials and Methods

### Patients and Tissue Samples

One hundred and seven human Non Small Cell Lung Carcinoma (NSCLC) and 25 associated normal lung parenchyma were included in this study. Tumors consisted of 54 adenocarcinoma (ADC) and 53 squamous cell carcinoma (SCC; Table 1). Tissue samples were collected from lung resection of lung tumors, and stored for scientific research in a biological resource repository (Centre de Ressources Biologiques, CHU Albert Michallon, Grenoble Hospital). National ethical guidelines were followed. All patients enrolled in this trial provided written informed consent. Tissue banking and research conduct was approved by the Ministry of Research (approval AC-2010-1129) and by the regional IRB (CPP 5 Sud Est). Tumor tissues and normal lung parenchyma taken at distance from the bulk of the tumor were immediately frozen and stored at −80°C until use. For histological classification, tumor samples were fixed in formalin, and diagnosis was made on paraffin-embedded material using the 2004 WHO classification of lung criteria [27]. For each case, one section from the most representative block was choosen. These sections always contained more than 70% of tumor cells.

**Figure 3:**
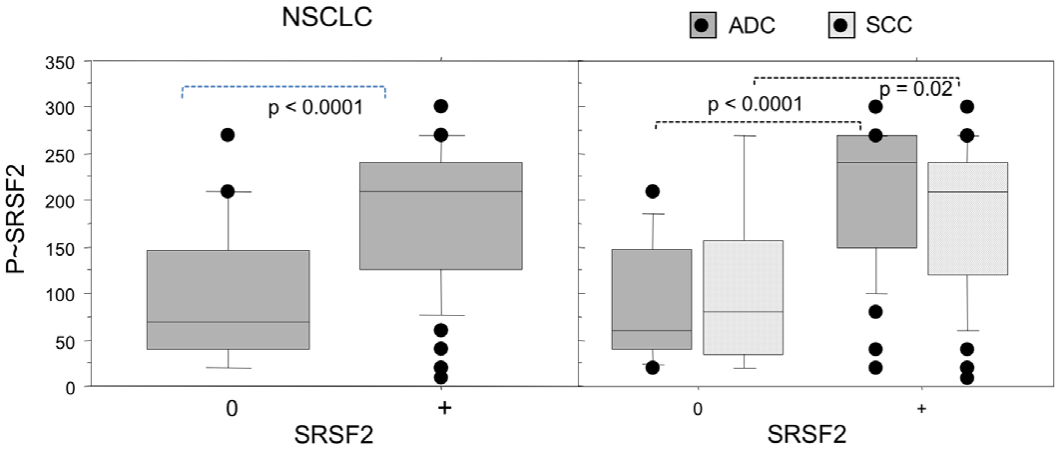
Relationship between SRSF2 overexpression and its phosphorylated status in NSCLC subtypes. Distribution of phospho-SRSF2 scores in tumors displaying either normal SRSF2 expression (class 0) or SRSF2 overexpression (class +), in all the tumors (left panels, NSCLC) and in histological subtypes (right panels, ADC and SCC). Statistical analysis was done using Mann-Whitney’s U test.

### Immunohistochemistry

Seven-micrometer-thick serial frozen sections were fixed with 3.7% paraformaldehyde for 10 min. A three-stage indirect immunoperoxidase technique was done either manually for SRSF1, SRSF2 and phosphorylated SRSF2, or on the Ventana autostainer (Ventana Medical International, Inc.) for SRPK1 and SRPK2. We used antibodies against SRSF1 (#32-4500, Invitrogen, dilution 1/1000), SRSF2 (SC35, #556363, BD Pharmingen, dilution 1/500), phospho-SRSF2 (#S4045, Sigma, dilution 1/500), SRPK1 (#611072, BD Biosciences, dilution 1/500) and SRPK2 (#611118, BD Biosciences, dilution 1/500). Incubation with the primary antibody at 4°C overnight (for manual technique) or for 1 hour at room temperature on Ventana autostainer was followed by exposition to the secondary biotinylated antibody, and then by the amplification system avidin-biotin complex. Negative control consisted in omission of the primary antibody and incubation with immunoglobulins of the same species and isotype.

**Figure 4:**
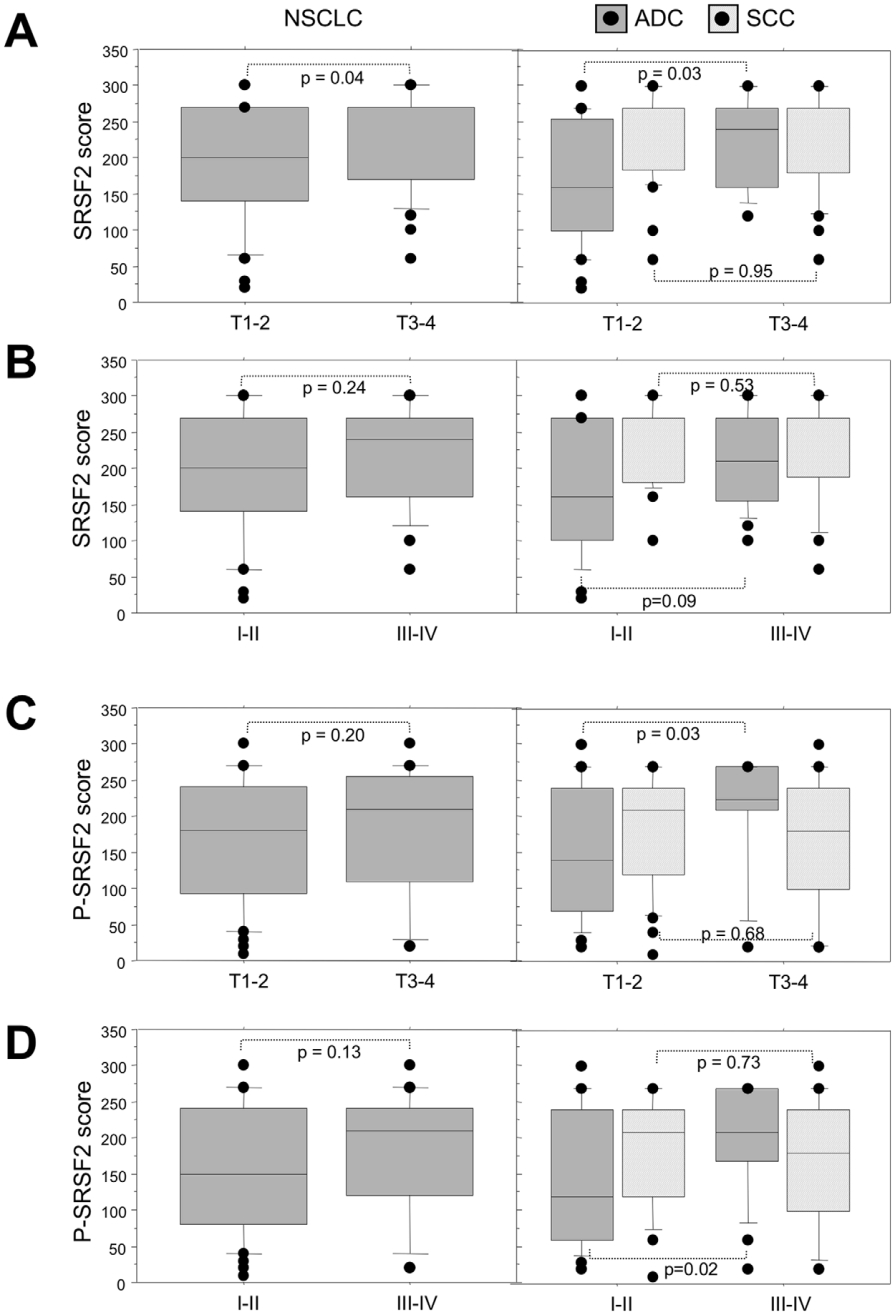
Expression of SRSF2 and its phosphorylated form according to the clinico-pathological parameters in NSCLC subtypes. Distribution of SRSF2 and phospho-SRSF2 scores according to the tumor size (A, C) and the stage (B, D), in all the tumors (left panels, NSCLC) and in histological subtypes (right panels, ADC and SCC). Statistical analysis was done using Mann-Whitney’s U test.

Immunostainings were evaluated independently by two pathologists who were blinded to all clinicopathological data (EB, SL). For immunostainings evaluation, a score (0–300) was established by multiplying the percentage of tumor labelled cells (0 to 100%) by the staining intensity (0, null; 1, low; 2, moderate; 3, strong). Scores obtained for alveolar type II pneumocytes and bronchial cells in normal lung tissues taken at distance from the tumor were considered as normal scores for adenocarcinoma and squamous cell carcinoma, respectively. Indeed, bronchial basal cells are considered as candidate cancer stem cells for squamous cell lung carcinoma, basaloids and Small Cell Lung Carcinoma while type II pneumocytes-CLARA cells are considered as bronchiolo alveolar carcinoma stem cells (BASC). According to median scores in normal tissues and to the distribution histograms (Figure S1), tumors were sub-divided in two classes for SRSF1 (class 1: low <100; class 2: high ≥100) and SRSF2 (class 1: low <150; class 2: high ≥150). For P-SRSF2, tumors were sub-divided in three classes (class 1: low <100; class 2: moderate ≥100; class 3: strong >175). For SRPK1 and SRPK2, distinct cut-off were chosen for adenocarcinoma and squamous cell carcinoma based on the different intensity of staining observed in alveolar type II pneumocytes (undetectable levels for both kinases) or bronchial cells (mean score of 50 for both kinases). According to these normal scores and to the distribution histograms (Figure S1), adenocarcinoma and squamous cell carcinoma were sub-divided in three classes as follows: class 1: low ≤50 or <100; class 2: moderate <200 or ≥100; class 3: strong ≥200 or >200, respectively.

**Table 3:** Immunohistochemical analysis of SRPK1 and SRPK2 proteins expression in non-small cell lung cancer according to histological subtype.

	SRPK1 expression			SRPK2 expression			
	Class 0	Class 1	Class 2	Class 0	Class 1	Class 2	P value
**ADC (%)**	2(4)	24(44)	28(52)	3(6)	33(61)	18(33)	<0.0001
**SCC (%)**	15(28)	16(30)	22(42)	17(32)	19(36)	17(32)	<0.0001
**NSCLC (%)**	17(16)	40(37)	50(47)	20(19)	52(48)	35(33)	<0.0001

Abbreviations: ADC, adenocarcinoma; SCC, squamous cell carcinoma; NSCLC, non-small cell lung carcinoma. Immunostaining scores were calculated by multiplying the number of labeled cells (0–100%) by the level of intensity (1–3). According to this, tumor samples were grouped into three classes (see ‘Materials and Methods’ section): class 0 (normal expression, as compared to normal lung), class 1 (moderate overexpression) and class 2 (high overexpression). Statistical analysis was done using Fisher’s exact test.

### Antibodies and Immunoblotting

Immunoblotting experiments were done as previously described [7]. The antibodies were anti-SRSF2 (4F11, Euromedex), anti-tubulin (TEBU, Le Perray-en-Yvelines), anti-AKT, anti-phospho-AKT(Ser473), anti-phospho-MAPK(ERK1/2) (Thr202/Tyr204) and anti-p44/42 MAPK from Cell Signaling (Ozyme, Saint Quentin Yvelines), anti-N-cadherin (32/N-Cadherin) and anti-E-cadherin (36/E-Cadherin) from BD Biosciences (Le Pont de Claix, France), anti-vimentin (clone V9) and anti-fibronectin (clone FN-15) from Sigma (Saint Quentin Fallavier, France).

### Cell Lines, Transfection, Treatments, Cytotoxic and Soft Agar Assays

H358, H1299 and H2170 cells were cultured in 5% CO_2_ at 37°C in RPMI-1640 medium supplemented with 10% (v/v) FCS. They were transfected either with a pcDNA3 empty vector or with a pcDNA3-SRSF1-Myc tagged vector using Fugene 6 reagent (Roche Diagnostic, France). H358 transfected cells were grown in RPMI-1640 medium containing 800 µg/ml geneticin (G418) for at least 4 weeks in order to select stable transfectants. Single colonies were isolated and expanded for further analyses. H1299 and H2170 transfected cells were selected during 6 days using 800 µg/ml G418 before analysis of EMT markers. U0126, wortmaninn, etoposide, paclitaxel and carboplatin were all purchased from Sigma (Saint Quentin Fallavier, France). Cytotoxic experiments were performed using the methylene blue colorimetric assay as previously described [7]. Anchorage-independent growth was determined by assaying colony formation in soft agar. Briefly, H358-Ctl and H358-SRSF1 cells were resuspended in RPMI containing 10% FCS and 0.3% agar noble (Difco™, BD Biosciences) and plated in quadriplicate on a firm 0.6% agar noble base in 12-well plates (15.000 cells/well). Colonies of cells were allowed to grow 21 days in a 37°C and 5% CO_2_ incubator. Colonies were then observed and counted under an inverted microscope (Zeiss Axiovert 100M). The colony formation assay was performed in triplicate.

**Figure 5:**
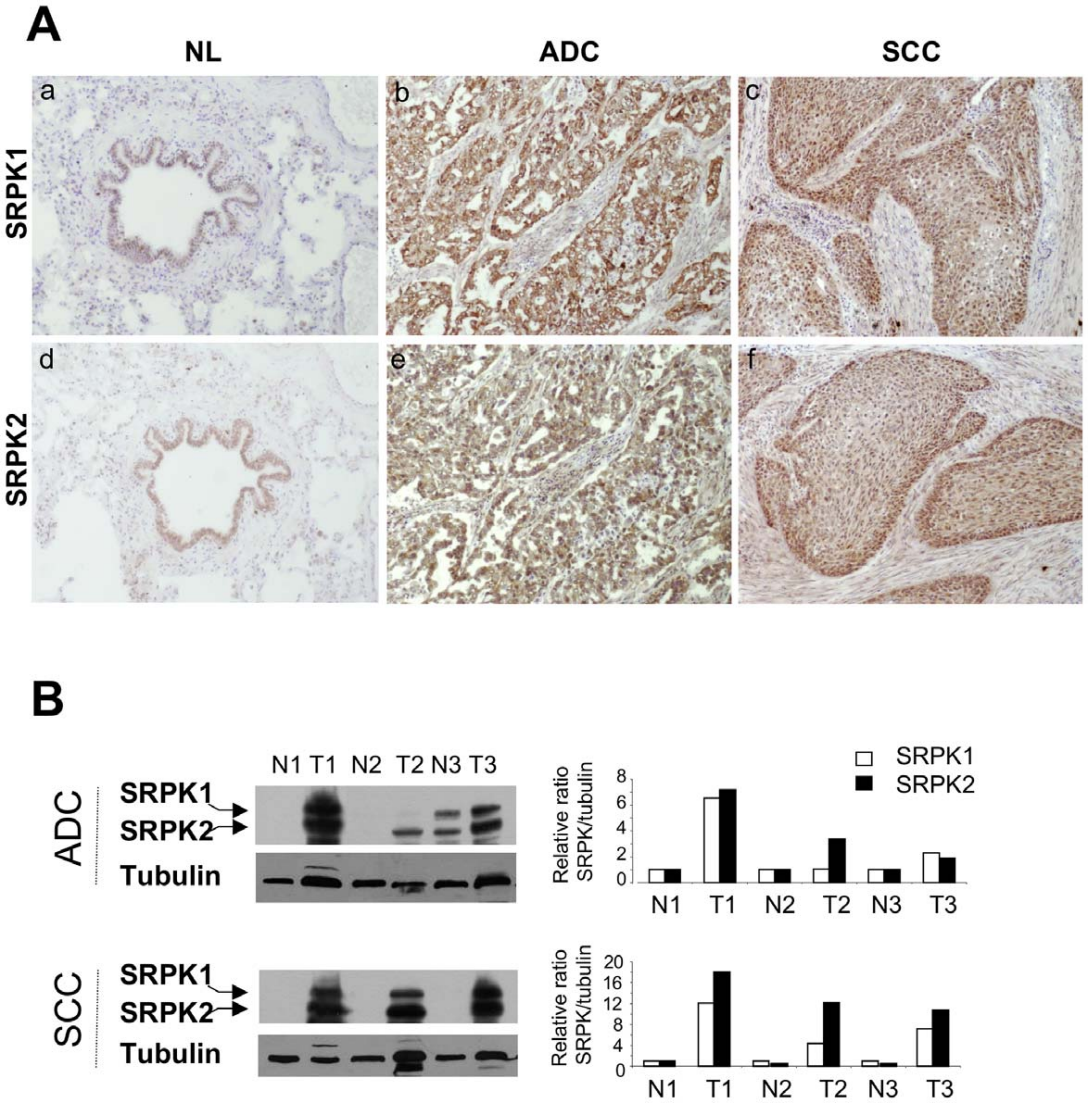
Expression of SRPK1 and SRPK2 proteins in NSCLCs. A, Representative immunostaining from frozen section of normal lung parenchyma and lung cancer tissue with anti SRPK1 (a, b, c) and anti SRPK2 (d, e, f) antibodies [(a, d) normal lung; (b, e) ADC; (c, f) SCC; immunoperoxidase and haematoxylin counterstaining]. B, Left panels: Representative western blots illustrating overexpression of SRPK1 and SRPK2 in lung tumors compared with their matched normal lung tissues. (NL, normal lung; ADC, adenocarcinoma; SCC, squamous cell carcinoma). Right panels: Densitometric analysis of western blot experiment was performed using Image J software. Each signal was quantified and the SRPK1/2:tubulin ratio was calculated in each case. The value 1 was arbitrarily assigned to the ratio obtained in normal tissues and a relative ratio was calculated for each tumor sample according to its normal tissue pair.

### RT–qPCR Analyses

Total RNA was extracted from normal and human lung tumors samples using RNeasy Mini Kit (Qiagen), according to the manufacturer’s instructions. RNA concentration and integrity was determined using NanoDrop ND-1000 spectrophotometer (Labtech). Quantitative real-time reverse transcript (RT)-qPCR was performed using the LightCycler® 480 Real-Time PCR system (Roche). One microgram of total RNA was subjected to cDNA synthesis with Superscript III First-Strand Synthesis SuperMix for qPCR (Invitrogen) and subsequently amplified during 45 PCR cycles using GoTaq® qPCR Master Mix (Promega). Primers used for the detection of *GAPDH* and *SRSF2* mRNAs were detailed previously [9]. Primers for amplification of *SRSF1*, *SRPK1* and *SRPK2* mRNAs were purchased from SA Biosciences (Qiagen). Relative gene expression was calculated for each sample, as the ratio of specific target gene to *GAPDH* gene (reference gene), thus normalizing the expression of target gene for sample to sample differences in RNA input.

**Figure 6:**
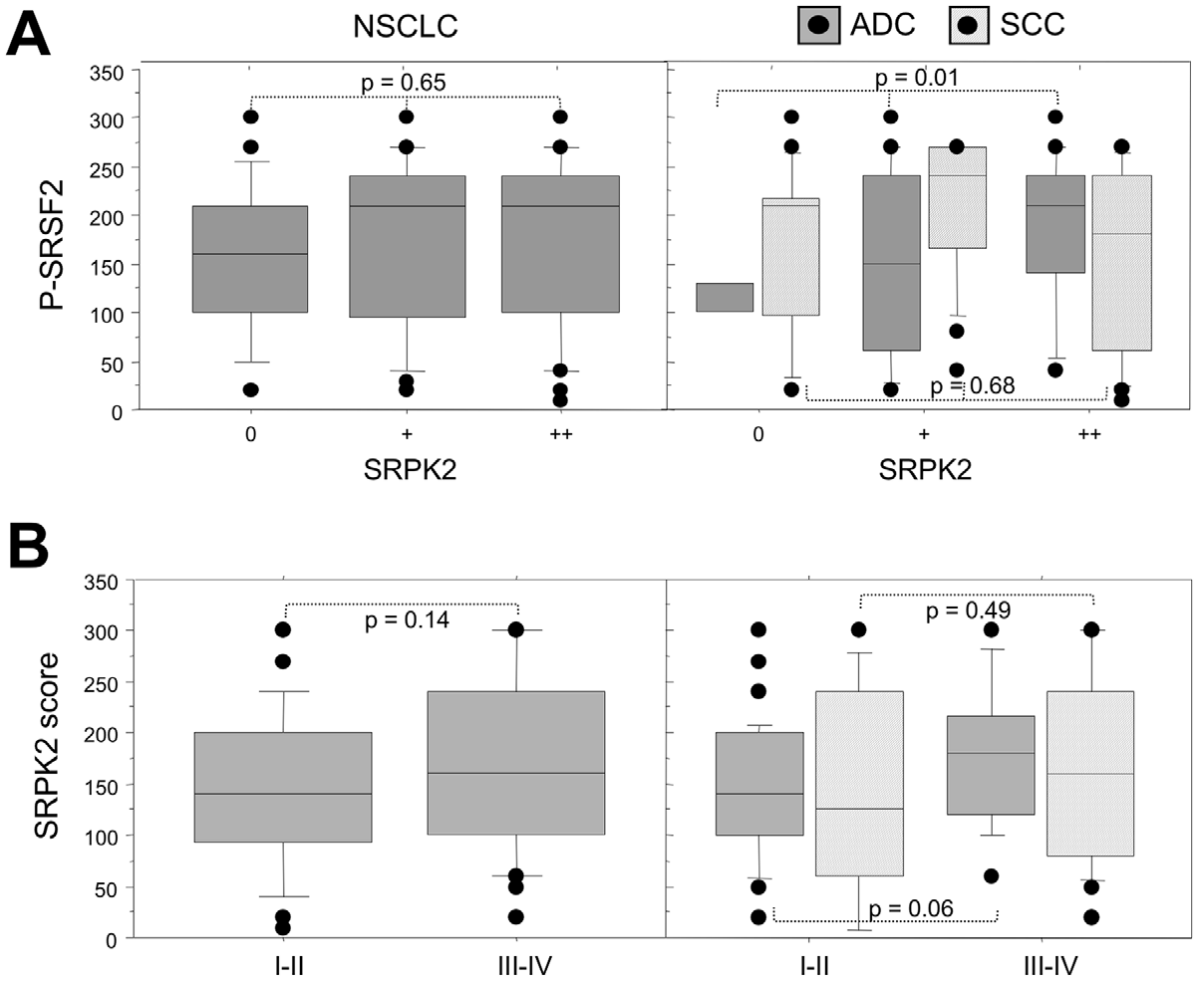
Relationship between SRPK2 protein expression and phospho-SRSF2 status in NSCLC subtypes. A, Distribution of phospho-SRSF2 scores in tumors displaying normal (class 0), moderate (class +) or overexpression (class ++) of SRPK2, in all the tumors (left panels, NSCLC) and in histological subtypes (right panels, ADC and SCC). B, Distribution of SRPK2 scores according to the tumor stage, in all the tumors (left panels, NSCLC) and in histological subtypes (right panels, ADC and SCC). Statistical analysis was done using Mann-Whitney’s U test.

### Statistical Analysis

The staining scores were compared in different categories using Fisher’s exact test and Mann-Whitney test. All tests were two-tailed and p values <0.05 were considered significant. The statistical analyses were done using Statview software (Abacus Concepts).

## Results

### Overexpression of SRSF1 Protein is Associated with Extensive Stage (III–IV) Tumors

We first analyzed the status of SRSF1 protein in our series of lung tumors. Of note, analysis of phospho-SRSF1 protein was not possible due to the lack of a specific anti-phospho-SRSF1 antibody. SRSF1 was slightly expressed in normal lung epithelium adjacent to tumor cells as well as in normal lung tissues localized at distance from lung cancer, with a faint nuclear staining on alveolar type II pneumocytes and a stronger nuclear staining on bronchial cells (mean score of 40 and 70 respectively, Figure 1A). Compared to these normal lung tissues, SRSF1 was overexpressed in 65% (70/107; p<0.0001 versus normal) of NSCLC, with almost the same frequency in adenocarcinoma (ADC; 34/54; 63%; p<0.0001 versus normal) and squamous cell carcinoma (SCC; 36/53; 68%; p<0.0001 versus normal) (Figure 1A & Figure S1; Table 2). SRSF1 belongs to a specific subset of SR proteins that shuttle continuously between the nucleus and the cytoplasm [28]. In NSCLC, SRSF1 accumulated predominantly in the nucleus thereby indicating that, besides its overexpression, modifications of SRSF1 sub-cellular distribution could also take place in lung tumors. In order to validate these IHC data, 6 of the 107 tumor samples and their matched normal lung tissues were analyzed for SRSF1 protein expression by western blotting (Figure 1B and data not shown). Again, we observed that SRSF1 protein was overexpressed in NSCLC compared to associated normal lung tissues. In order to test whether SRSF1 protein overexpression correlates with *SRSF1* mRNA increase, we performed RT-QPCR in a series of 25 NSCLCs and their matched normal lung. We did not find any correlation between SRSF1 mRNA and protein levels (Figure S2).

**Figure 7:**
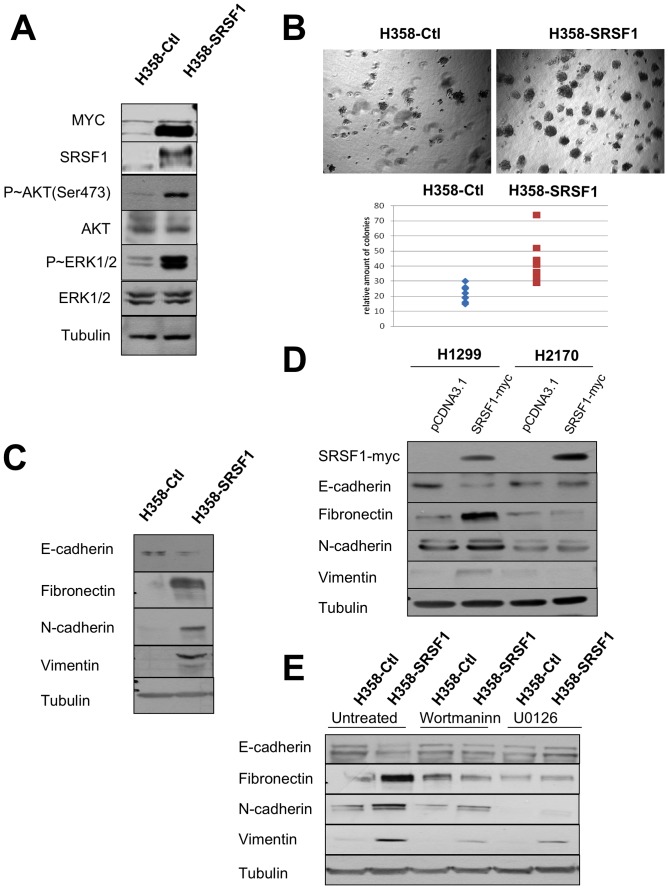
SRSF1 overexpression confers a more aggressive phenotype. Western blot analyses (A, C, E) and soft agar assays (B) were performed in H358-Ctl and H358-SRSF1 cells that were cultured at the same passage. (A) Expression of SRSF1 and activation of AKT/ERK signaling pathways were analyzed by western blotting. Tubulin was used as a loading control. (B) Upper panels: representative images of colonies in soft agar. Magnification, ×500. Lower panel: relative amounts of colonies in eight representative fields. Three independent experiments were performed in quadriplicate. (C) Western blot analysis of epithelial and mesenchymal markers. Tubulin was used as a loading control. (D) H1299 and H2170 cells were transfected either with control pcDNA3.1 or with myc-tagged SRSF1 plasmid. Transfected cells were selected during 6 days with G418 (800 µg/ml) and western blot analyses were performed using the indicated antibodies. Tubulin was used as a loading control. (E) H358-Ctl and H358-SRSF1 cells were cultured for 72 hours in the presence or absence of 500 nM wortmaninn or 10 µM U0126 as indicated. Expression of epithelial (E-cadherin) and mesenchymal (fibronectin, N-cadherin, vimentin) markers was analyzed by western blotting. Tubulin was used as a loading control.

To further characterize the role of SRSF1 during lung carcinogenesis, we analyzed the relationships linking the IHC data and some clinicopathological features (Figure 2). High levels of SRSF1 expression were associated with the presence of metastases at distance (M+, p = 0.001) and extensive stage (III/IV, p = 0.002) in NSCLC. When histological sub-types were distinguished, a correlation between the presence of metastases (p = 0.004) and extensive stage III/IV (p = 0.006) was found in ADC only (Figures 2C, 2D and Figure S3). Taken together, these results demonstrate that SRSF1 protein is overexpressed in a vast majority of NSCLC compared to normal lung tissues and is associated with criteria of tumor invasiveness in lung adenocarcinoma.

### Expression of SRSF2 and Phospho-SRSF2 Proteins is Correlated in NSCLC

Next, we investigated the status of SRSF2 and its phosphorylated form (P-SRSF2) in the same series of tumor samples by IHC. The anti-phospho-SRSF2 antibody was specific of phosphorylated SRSF2 as it strongly detected SRSF2 but not SRSF1 protein in cellular models stably overexpressing each protein (Figure S4). A moderate nuclear staining of SRSF2 and P-SRSF2 proteins was observed in alveolar type II pneumocytes (mean scores of 80 and 46, respectively), whereas bronchial cells exhibited a stronger nuclear staining (mean scores of 160 and 70, respectively) (Figure 1A). Compared to these normal lung epithelia, high levels of SRSF2 nuclear staining were observed in 83 out of 107 (78%) NSCLC (p<0.0001 versus normal; Table 2). According to each histological subtype, overexpression of SRSF2 protein was detected in 35/54 ADC (65%; p<0.0001 versus normal) and 48/53 SCC (91%; p<0.0001 versus normal) (Table 2; Figure 1A and Figure S1). Similarly, and compared to normal lung tissues, a moderate (class 1, scores ranging from 100 to 175) or strong (class 2, scores ranging from 176 to 300) P-SRSF2 immunostaining was observed in 82/107 (77%) NSCLC, including 40/54 (74%) ADC and 42/53 (79%) SCC (p<0.0001 versus normal; Table 2 and Figure 1A). Interestingly, SRSF2 and P-SRSF2 levels were highly correlated in NSCLC (p<0.0001), ADC (p<0.0001) and SCC (p = 0.02) (Figure 3). In order to validate these IHC results, SRSF2 protein expression was studied by western blotting in 6 of the 107 tumor samples and their matched normal lung tissues (Figure 1B). Again a good concordance was found between both techniques. Overall, these results demonstrate that both SRSF2 and P-SRSF2 proteins are overexpressed and correlate in a vast majority of NSCLC. Of note, when *SRSF2* mRNA levels were analyzed in the same samples than SRSF1, no correlation was found between SRSF2 mRNA and protein levels (Figure S2).

**Figure 8:**
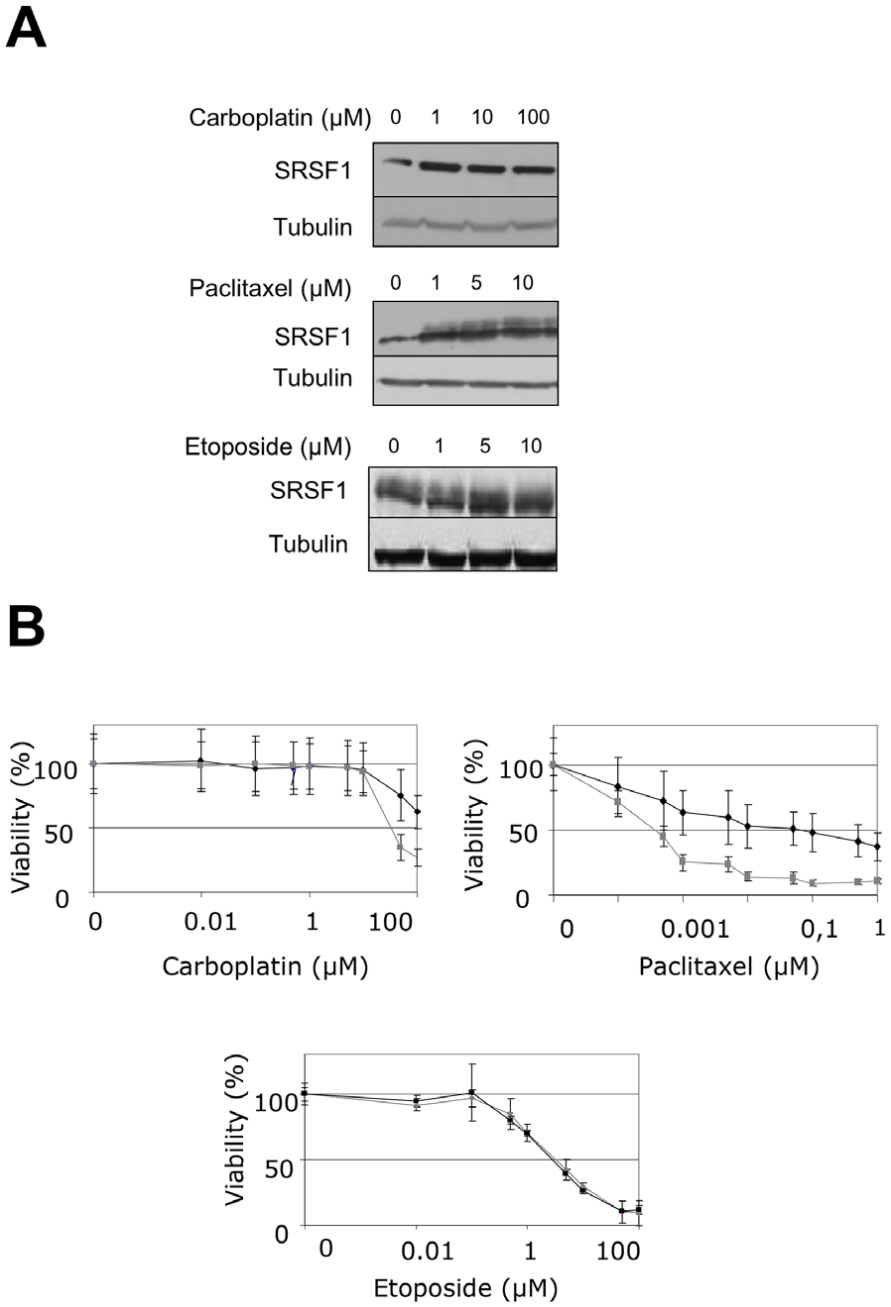
SRSF1 overexpression increases resistance to carboplatin and paclitaxel. (A) H358 cells were treated for 24 hours with increasing amounts of etoposide, carboplatin or paclitaxel as indicated. SRSF1 protein level was analyzed by western blotting. Tubulin was used as a loading control. (B) 96-hours cell viability assays were performed in H358-Ctl cells (grey symbols) or H358-SRSF1 clones (black symbols) treated or not with increasing amounts of carboplatin, paclitaxel or etoposide. Results are expressed as the percentage of survival cells compared to untreated cells. Mean value of three independent experiments ± standard deviation performed in triplicate are presented.

To go further, we analyzed the relationships linking SRSF2 and P-SRSF2 status and some of the clinicopathological characteristics. High scores of either SRSF2 or P-SRSF2 were associated with larger size tumors (T3–T4) in ADC (p = 0.03; Figure 4A & 4C). Furthermore, high levels of P-SRSF2 correlated with extensive stage (III–IV) in ADC (p = 0.02; Figure 4D). These results indicate that accumulation of P-SRSF2 is associated with a more aggressive phenotype in adenocarcinoma.

### Both SRPK1 and SRPK2 Kinases are Overexpressed in NSCLC

We recently reported that the SRPK1 and SRPK2 kinases control SRSF2 phosphorylation in cellular models derived from NSCLC [10]. Therefore, we evaluated the status of these kinases by IHC in our series of primary tumors. SRPK1 and SRPK2 proteins were faintly expressed in normal bronchial cells (mean score of 30 and 50, respectively) with a nuclear and cytoplasmic pattern, but were undetectable in alveolar type II pneumocytes (Table 3 and Figure 5). Compared to these normal lung tissues, SRPK1 was upregulated in 90/107 (84%), 52/54 (92%) and 38/53 (72%) NSCLC, ADC and SCC, respectively (p<0.0001 versus normal; Table 3). In the same way, overexpression of SRPK2 was observed in 87/107 (81%), 51/54 (94%) and 36/53 (68%) NSCLC, ADC and SCC respectively (Table 3; p<0.0001 versus normal). Both kinases exhibited a nuclear and cytoplasmic pattern. These results provide the first evidence that SRPK1/SRPK2 kinases are overexpressed in NSCLC. Again, a good concordance was observed between IHC and western blot data (Figure 5B). Of note, as for *SRSF1* and *SRSF2*, no correlation was found between SRPK1/SRPK2 proteins and mRNA levels in tumors (Figure S2). These data suggest that post-transcriptional mechanisms control the expression level of splicing regulators in human lung tumors. Interestingly, a direct correlation was detected between P-SRSF2 and SRPK2 stainings in ADC (p = 0.01; Figure 6). By contrast, no relationship was found between SRPK1 and P-SRSF2 expression, whatever the histological sub-type (data not shown). These data suggest that SRPK2 is the main kinase phosphorylating SRSF2 in ADC, consistent with our previous results obtained in cell lines [10]. Finally, when clinicopathological parameters were analysed as regard to either SRPK1 or SRPK2 status, high levels of SRPK2 tended to be associated with extensive stage in ADC (p = 0.06, Figure 6).

### SRSF1 Overexpression Promotes a More Aggressive Phenotype in Adenocarcinoma

We recently demonstrated that SRSF2 overexpression controls apoptosis and contributes to the response of NSCLC cell lines to cisplatin [10]. To further study the consequences of increased SRSF1 expression in NSCLC, the human lung adenocarcinoma cell line H358 was stably transfected with an expression vector encoding a myc-tagged SRSF1 protein and several clones overexpressing myc-SRSF1 were obtained. The results of a representative clone are presented. Compared to control cells, SRSF1-overexpressing cells accumulated P-AKT(Ser473) and phospho p42/p44MAPK proteins (Figure 7A). In addition, they generated significantly more colonies in soft agar (Figure 7B). SRSF1-overexpressing cells also evidenced an epithelial to mesenchymal transition (EMT) as demonstrated by the loss of epithelial markers such as E-cadherin and the acquisition of mesenchymal markers such as vimentin, fibronectin and N-cadherin (Figure 7C). In order to extend these data, two other cell lines, namely the H1299 adenocarcinoma and the H2170 squamous carcinoma cells, were transfected either with a control plasmid or with a plasmid encoding a myc-tagged SRSF1 protein and subjected to G418 selection for 6 days. Acquisition of mesenchymal markers such as N-cadherin or vimentin was detected in H1299 cells overexpressing SRSF1 (Figure 7D), thereby confirming the results obtained in H358 stable clones. In contrast, such EMT was not detected in H2170 cells overexpressing SRSF1 (Figure 7D). In addition, EMT was partially reversed when H358 SRSF1-overexpressing cells were cultured in presence of wortmaninn or U0126, two pharmacological inhibitors targeting AKT and MEK/ERK signaling pathways respectively (Figure 7E). Taken together, these results demonstrate that SRSF1 overexpression promotes a more aggressive phenotype in lung adenocarcinoma but not in lung squamous carcinoma cell lines. They are consistent with the IHC results showing high levels of SRSF1 in extensive stage ADC only (p = 0.006).

### SRSF1 Overexpression Increases Resistance to Chemotherapy

Acquisition of an EMT phenotype has been associated with resistance to chemotherapy in NSCLCs [29]. Therefore, we tested whether SRSF1 overexpression affects the sensitivity of lung tumor cells to chemotherapeutic agents. We observed that SRSF1 protein accumulates in H358 cells treated with carboplatin and paclitaxel but not with etoposide (Figure 8A). In addition, H358 cells stably overexpressing SRSF1 were more resistant to carboplatin and paclitaxel than control cells (Figure 8B). This was not an unspecific effect related to SRSF1 overexpression since both control and SRSF1-overexpressing cells exhibited similar sensitivity to etoposide (Figure 8B). Altogether, these data indicate that SRSF1 is a component of the lung tumor cells response to chemotherapies.

## Discussion

A growing body of evidence indicates that SR proteins are directly involved in the process of carcinogenesis, acting as proto-oncogenes [25], [30] or regulating splicing and activity of proto-oncogenes [29], tumor suppressors [25] or apoptotic regulators [30]. However to date, only a few studies have investigated the status of these proteins and their regulators *in situ* in human tumors. In this study, we provide the first evidence that SRSF1, SRSF2, P-SRSF2, as well as the SR-phosphorylating kinases SRPK1 and SRPK2 are up-regulated in NSCLC. These data therefore indicate that a global deregulation of critical splicing regulators likely contributes to lung tumorigenesis.

Upregulation of SRSF1 and SRSF2 proteins has been shown in a large variety of carcinoma, including renal, breast, ovarian, cervical, colon and pancreatic cancers [30]–[36]. By contrast, the status of SRPK1 or SRPK2 remains poorly investigated in human tumors [37]–[39]. Recently, Montuenga’s group reported that SRSF1 is overexpressed in lung adenocarcinoma in which it controls the expression of survivin, an anti-apoptotic protein [26]. Here, we demonstrate that not only SRSF1 but also SRSF2, SRPK1 and SRPK2 proteins are overexpressed in both adenocarcinoma and squamous cell lung carcinoma. Nuclear localization of SR proteins is required for their splicing activity and is dependent on their phosphorylation by SR kinases [28], [40]. Unfortunately, we could not investigate the phosphorylated status of SRSF1 due to the lack of a specific antibody directed against phospho-SRSF1. However, we found a strong correlation between SRSF2 and P-SRSF2 IHC scores in both ADC (p<0.0001) and SCC (p = 0.02), supporting the notion that SRSF2 mainly accumulates under an hyper-phosphorylated form in lung tumors, especially in ADC. In addition, we showed that P-SRSF2 and SRPK2 are directly correlated, suggesting that SRPK2 is the main kinase phosphorylating SRSF2 in this histological sub-type. These data are consistent with our previous study demonstrating that SRPK1 and SRPK2 control SRSF2 phosphorylation in cellular models derived from ADC [10]. However, we cannot exclude the possibility that other SR kinase(s) such as Clk/Sty also contribute to SRSF2 phosphorylation in NSCLCs. We also observed that SRSF1 mainly accumulates in the nucleus of lung tumor cells. Since SRSF1 has been shown to continuously shuttle between the nucleus and the cytoplasm, its nuclear accumulation could reflect its mislocalisation and/or deregulated activity in lung tumors. In agreement with such a notion, very recently the nuclear functions of SRSF1 were shown to be involved in the transformation of mammary epithelial cells in cooperation with the myc oncogene [30]. Overall, our results support the idea that variations in expression, localization and/or activity (through phosphorylation) of SRSF1 and SRSF2 proteins take place during lung cancer progression. Interestingly, and despite high IHC scores, we did not observe a correlation between *SRSF1*, *SRSF2*, *SRPK1* and *SRPK2* mRNA and protein levels in the vast majority of tumors compared to normal lung tissues. These results indicate that post-transcriptional regulations merely account for the accumulation of SRSF1, SRSF2, SRPK1 and SRPK2 proteins in NSCLCs.

ADC and SCC are distinct entities in terms of gene expression, tumor response to therapy or clinical outcome [41]–[43]. In this study, we do not observe a relationship between SRSF1, SRSF2, P-SRSF2 status and clinico-pathological parameters in SCC, although these proteins are highly overexpressed. In contrast, we demonstrate that high levels of P-SRSF2 correlate with larger tumor size and extensive stage in ADC. Therefore, maintenance of SRSF2 in an hyper-phosphorylated form could lead to a more aggressive phenotype in this histological sub-type. This is consistent with the role of SRSF2 in cell proliferation during mammalian organogenesis [44]. However, we recently demonstrated that SRSF2 is required for cisplatin-induced apoptosis in human lung cell lines derived from ADC [10] and showed that SRSF2 overexpression *per se* induces apoptosis (Moysan E, unpublished data). It is thus tempting to speculate that NSCLCs have counteracted this SRSF2-dependent apoptosis. We also show that high levels of SRSF1 are associated with extensive stage in ADC (p = 0.004; Figure S3), suggesting that SRSF1 has a potential role during the metastatic progression of ADC. In agreement with such hypothesis, we demonstrate that H358 adenocarcinoma cells overexpressing SRSF1 acquire a more invasive phenotype as illustrated by hyperactivation of the oncogenic AKT and ERK signaling pathways, increased capacity to form colony in soft agar and acquisition of mesenchymal markers such as vimentin and N-cadherin. It was previously shown in mammary and gastric carcinoma cell lines that overexpression of SRSF1 activates the epithelial to mesenchymal transition (EMT) and induces a more invasive phenotype [31], [45]. Therefore, our data extend to lung carcinoma the aggressive property of SRSF1 protein. We also demonstrate that SRSF1 overexpression decreases the sensitivity of H358 cells to carboplatin and paclitaxel, two chemotherapeutic agents widely used in clinic. It was recently shown that increased EMT markers expression in tumor specimens obtained from patients with NSCLC correlates with reduced sensitivity to cisplatin and paclitaxel [29]. Taken together, these data indicate that SRSF1-induced EMT plays a role in the resistance to chemotherapy. We recently provided evidence that SRSF2 accumulates in NSCLC cell lines treated with cisplatin and is required for induction of apoptosis in this setting [10]. Therefore, SRSF1 and SRSF2 proteins appear to play opposite roles during the response of NSCLC to genotoxic stresses.

In summary, we provide evidence that high scores of SRSF1, SRSF2, and P-SRSF2 proteins correlate with more aggressive features in ADC but not in SCC, thereby suggesting that these proteins do not predict same outcome in both histological sub-types. In addition, we demonstrate for the first time that the SR phosphorylating kinases SRPK1 and SRPK2 are up-regulated in NSCLC. Additional studies are now required to better characterize at which step of the lung carcinogenesis process the deregulation of SR and SRPK proteins occurs, to elucidate the regulatory pathways that control their expression and activation, and to identify some of their target genes. We recently showed that inhibition of SRPK1 enhances sensitivity of lung cancer cell lines to cisplatin [10]. It has also been reported that the knock-down of SRPK1 in pancreatic carcinoma increases sensitivity to gemcitabine and cisplatin [37], [38]. As overexpression of SRPK appears as a typical feature of lung tumor cells, these results open a promising potential for SRPK inhibitors as anti-cancer agents. In the future, pharmacological treatments that target aberrant pre-mRNA splicing will lead to the development of new anti-cancer therapeutic strategies.

## Supporting Information

Figure S1 **Distribution of SRSF1, SRSF2, Phospho-SRSF2, SRPK1 and SRPK2 stainings across 54 ADC and 53 SCC samples.** (ADC, adenocarcinoma; SCC, squamous cell carcinoma).
(TIF)

Figure S2 **mRNA levels of SRSF1, SRSF2, SRPK1 and SRPK2 in normal lung and NSCLC.** RT-qPCR analysis of mRNA levels in 25 NSCLC and associated normal lung parenchyma samples. GAPDH was used as an internal control. Relative gene expression was calculated for each sample, as the ratio of target gene to GAPDH gene (reference gene), thus normalizing the expression of target gene for sample to sample differences in RNA input. For each couple normal/tumor couple, the mRNA level obtained in normal lung was arbitrarily assigned the value of 1.
(TIF)

Figure S3 **SRSF1 expression according to pTNM stages in NSCLC.** Distribution of SRSF1 scores in all the tumors (left panels, NSCLC) and in histological subtypes (right panels, ADC and SCC). Statistical analysis was done using Kruskal-Wallis test.
(TIF)

Figure S4 **Western blot analysis of P-SRSF2 protein expression in H358 cells overexpressing SRSF2 (A) or SRSF1 (B) protein.** **A.** SRSF2 and its phosphorylated form are overexpressed in H358 cells stably transfected with a Tet-responsive SRSF2 vector and cultured in the presence (+) or absence (−) of 1 µg/ml doxycyclin. The results obtained with two different clones are presented. **B.** The anti-phospho SRSF2 antibody used in IHC does not recognize SRSF1 protein even when overexpressed in stable H358 clone transfected with a SRSF1 encoding vector. Tubulin was used as a loading control.
(TIF)
