# Supplementary figures and images for: Abnormal Expression of the Pre-mRNA Splicing Regulators SRSF1, SRSF2, SRPK1 and SRPK2 in Non Small Cell Lung Carcinoma
Source: PLoS One. 2012 Oct 10;7(10):e46539. doi: 10.1371/journal.pone.0046539 (PMC3468597; doi:10.1371/journal.pone.0046539)

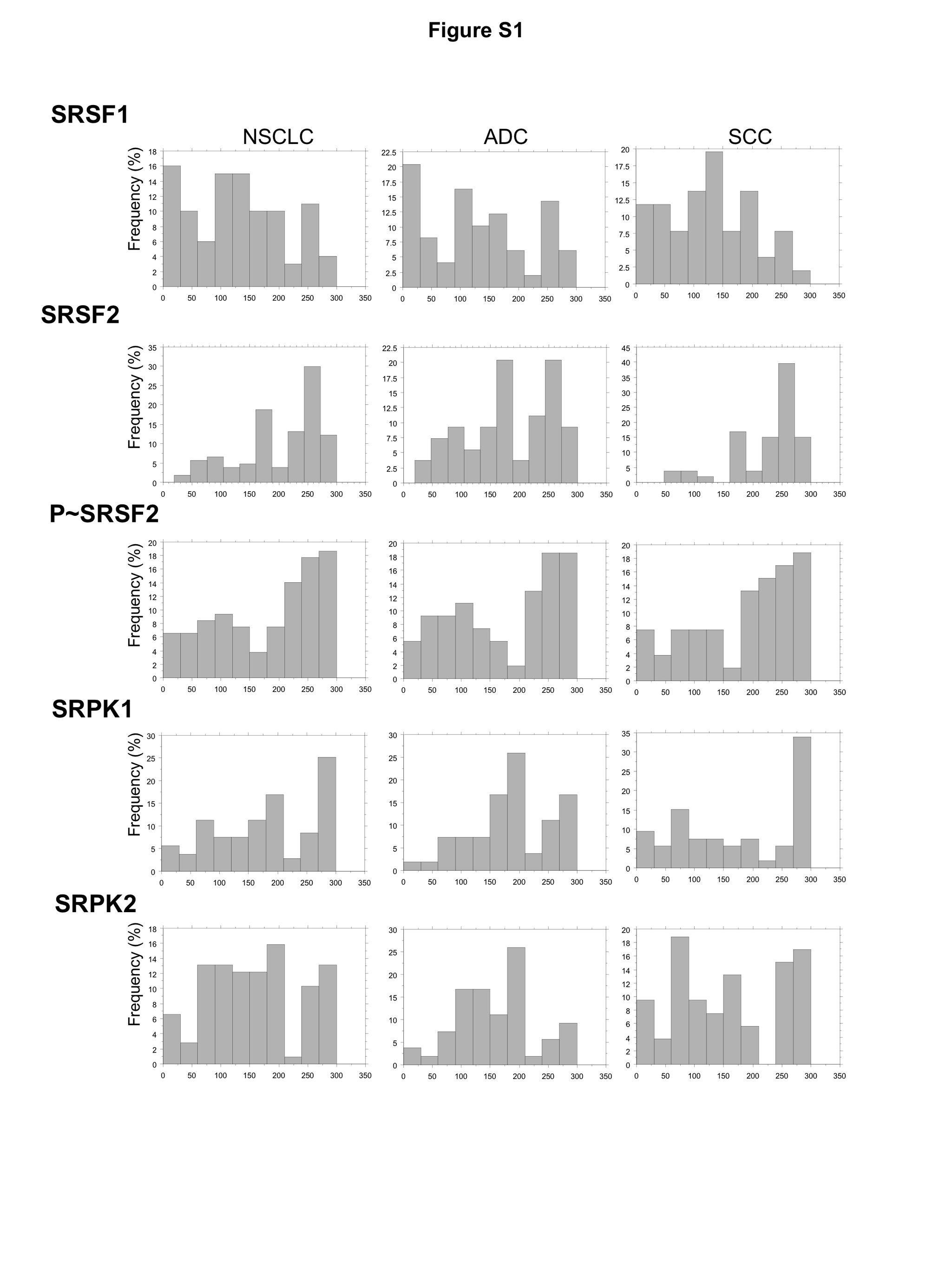

Supplement: Figure S1 — Distribution of SRSF1, SRSF2, Phospho-SRSF2, SRPK1 and SRPK2 stainings across 54 ADC and 53 SCC samples. (ADC, adenocarcinoma; SCC, squamous cell carcinoma). (TIF) [file pone.0046539.s001.tif]

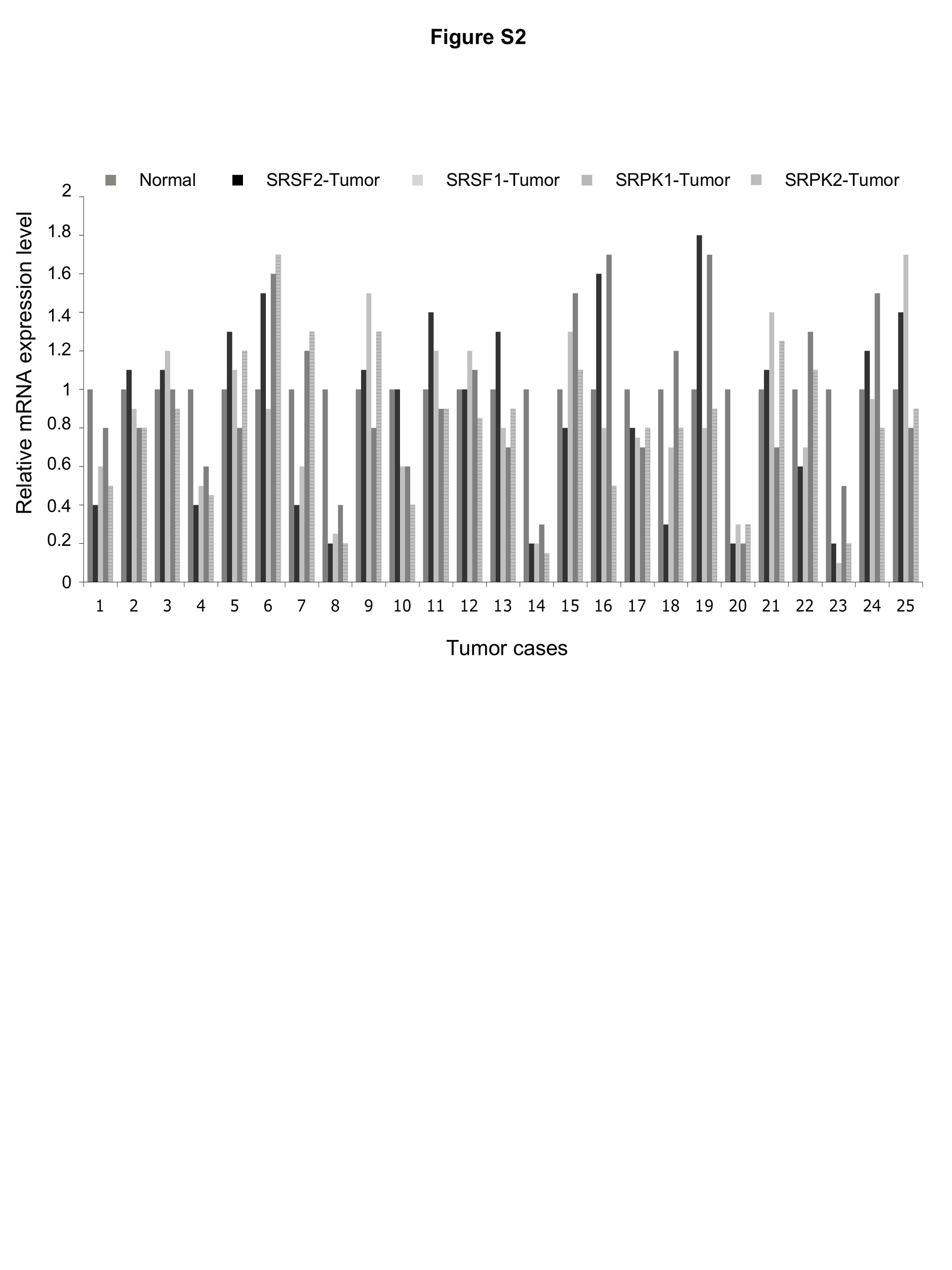

Supplement: Figure S2 — mRNA levels of SRSF1, SRSF2, SRPK1 and SRPK2 in normal lung and NSCLC. RT-qPCR analysis of mRNA levels in 25 NSCLC and associated normal lung parenchyma samples. GAPDH was used as an internal control. Relative gene expression was calculated for each sample, as the ratio of target gene to GAPDH gene (reference gene), thus normalizing the expression of target gene for sample to sample differences in RNA input. For each couple normal/tumor couple, the mRNA level obtained in normal lung was arbitrarily assigned the value of 1. (TIF) [file pone.0046539.s002.tif]

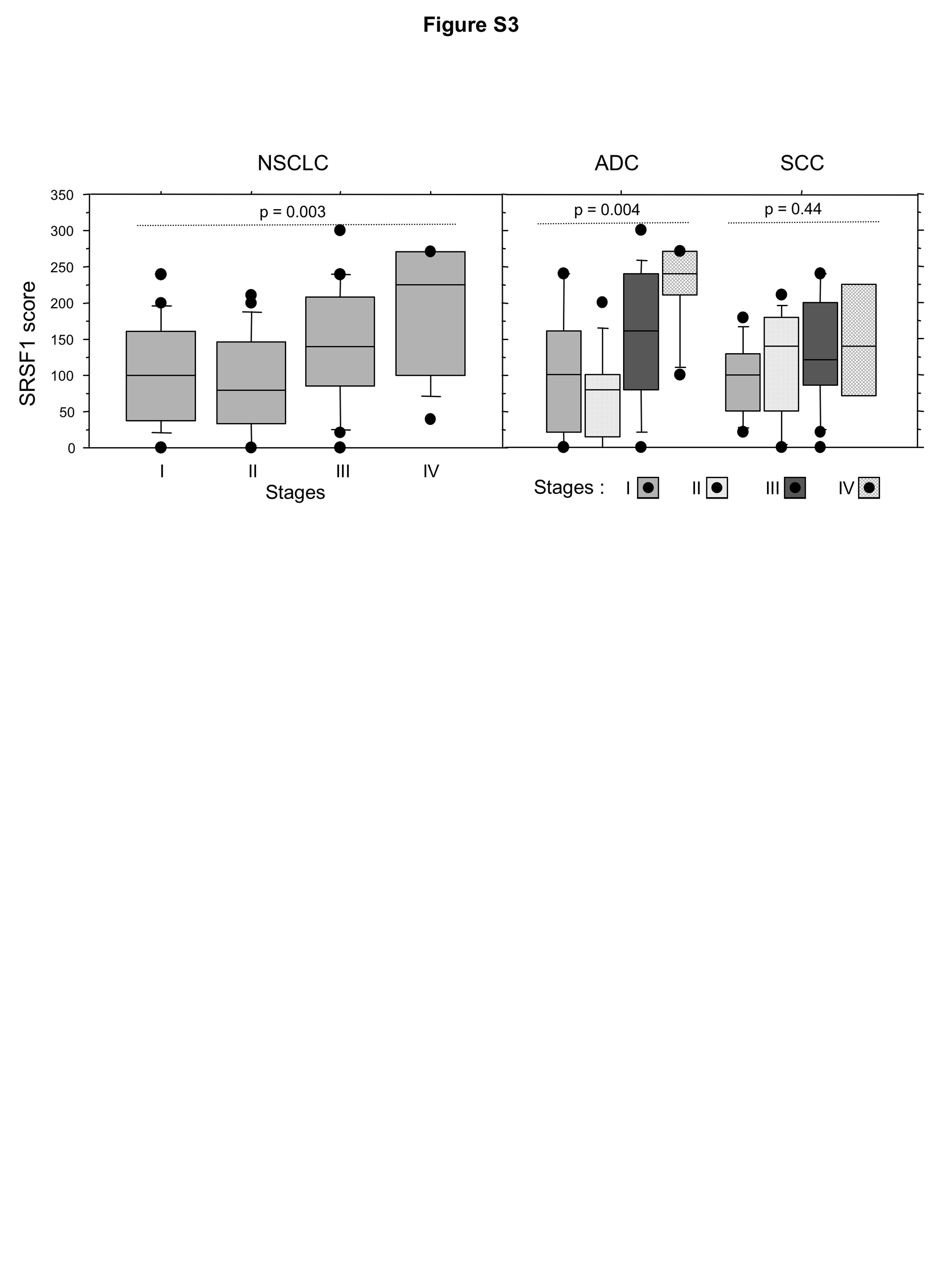

Supplement: Figure S3 — SRSF1 expression according to pTNM stages in NSCLC. Distribution of SRSF1 scores in all the tumors (left panels, NSCLC) and in histological subtypes (right panels, ADC and SCC). Statistical analysis was done using Kruskal-Wallis test. (TIF) [file pone.0046539.s003.tif]

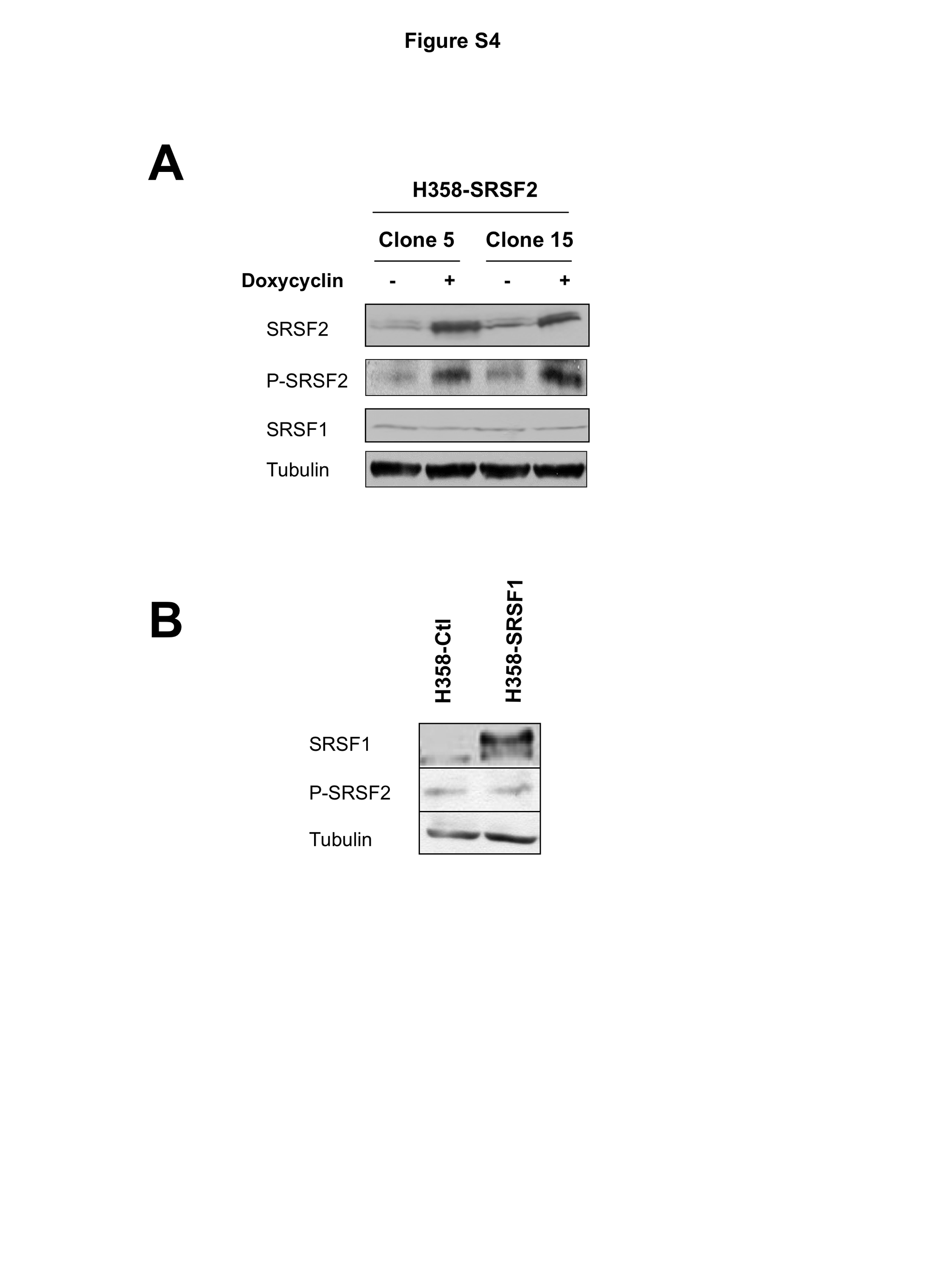

Supplement: Figure S4 — Western blot analysis of P-SRSF2 protein expression in H358 cells overexpressing SRSF2 (A) or SRSF1 (B) protein. A. SRSF2 and its phosphorylated form are overexpressed in H358 cells stably transfected with a Tet-responsive SRSF2 vector and cultured in the presence (+) or absence (−) of 1 µg/ml doxycyclin. The results obtained with two different clones are presented. B. The anti-phospho SRSF2 antibody used in IHC does not recognize SRSF1 protein even when overexpressed in stable H358 clone transfected with a SRSF1 encoding vector. Tubulin was used as a loading control. (TIF) [file pone.0046539.s004.tif]
